# The Montreal Veterinary Medical Association

**Published:** 1897-11

**Authors:** W. K. Wallis

**Affiliations:** Secretary-Treasurer


Secretary.

### VETERINARY MEDICAL ASSOCIATION OF NEW JERSEY.

Secretary.

### THE MONTREAL VETERINARY MEDICAL ASSOCIATION.

THE regular meeting of the Association was held on the evening of October 14, 1897, in the library, the retiring President, Professor Baker, occupying the chair. After roll-call and the reading of minutes of the previous meeting, which were accepted, the election of officers for the ensuing year, which had been postponed, took place, with the following result: President, Prof. Charles McEachran; First Vice-President, Prof. M. C. Baker; Second Vice-President, Dr. Martin; Secretary-Treasurer, W. B. Wallis; Librarian, J. P. Stanton. The retiring President then made a few farewell remarks, pointing out the advantages of such societies, especially to students, in educating them how to properly express themselves in public, and also that the reading of essays necessitated a man getting up his subject exceptionally well, in order to properly defend his paper. The newly elected President then took the chair, and after expressing his appreciation of the honor done him, urged upon the members the value of practical work and of the experimental committee in making careful reports of the action of drugs.

Mr. Lambert, being called upon by the President, read a practical though somewhat brief essay on "The Nature and Treatment of Inflammation." Treating his subject from a clinical rather than a theoretical point of view, he explained the reactive phenomena occurring in nature during the process by some original illustrations.

In the argument which then followed it was suggested that the benefit derived from applying counter-irritation to a diseased articulation might in part be due to the prolonged rest and degree of inability such an agent enforced on the part.

Mr. Wallis then reported a severe case of pneumonia in a race-horse, his object being to advocate the use of some preventive treatment, during convalescence, against the disastrous whistling and roaring that were so frequently commemorative of diseases of the respiratory organs situated in the thoracic cavity.

For this purpose he suggested a course of potassium iodide and nux vomica, the former to hasten the absorption of inflammatory products, while the latter might correct any defective nervous power.

The President then appointed Messrs. Paquin and Spanton as essayists for the following meeting, and Mr. Lambert to report a case.

Messrs. Bell and Spanton were appointed as acting members on the Experimental Committee.

There being no further business, the meeting then adjourned.

W. K. WALLIS,
Secretary-Treasurer.

